# Differences in perceptual latency estimated from judgments of temporal order, simultaneity and duration are inconsistent

**DOI:** 10.1068/i0675

**Published:** 2014-11-11

**Authors:** Daniel Linares, Alex O. Holcombe

**Affiliations:** Laboratoire Psychologie de la Perception, Université Paris Descartes, Paris, France; e-mail: danilinares@gmail.com; School of Psychology, University of Sydney, Sydney, New South Wales, Australia; e-mail: alex.holcombe@sydney.edu.au

**Keywords:** TOJ, SJ, duration, inconsistent, time, perception

## Abstract

Differences in perceptual latency (*ΔL*) for two stimuli, such as an auditory and a visual stimulus, can be estimated from temporal order judgments (TOJ) and simultaneity judgments (SJ), but previous research has found evidence that *ΔL* estimated from these tasks do not coincide. Here, using an auditory and a visual stimulus we confirmed this and further show that *ΔL* as estimated from duration judgments also does not coincide with *ΔL* estimated from TOJ or SJ. These inconsistencies suggest that each judgment is subject to different processes that bias *ΔL* in different ways: TOJ might be affected by sensory interactions, a bias associated with the method of single stimuli and an order difficulty bias; SJ by sensory interactions and an asymmetrical criterion bias; duration judgments by an order duration bias.

## 1 Introduction

Perceiving a stimulus takes time and this perceptual latency, *L*, likely depends on the sensory feature. For example, auditory stimuli may be processed faster than visual stimuli. Knowing how much longer it takes to perceive a stimulus *A* relative to a stimulus *B*—the difference in perceptual latency, *ΔL*—may help the project of understanding what kind of neural activity causes perception (Krekelberg & Lappe, 2001; Zeki, 2003).

*ΔL* is sometimes estimated by subtracting the behavioural response time to stimulus *B* from the response time to stimulus *A* (Amano, Johnston, & Nishida, 2007; Aschersleben & Musseler, 1999; Di Luca, Machulla, & Ernst, 2009; Kopinska & Harris, 2004; Navarra, Hartcher-O'Brien, Piazza, & Spence, 2009; Neumann & Niepel, 2004; Nishida & Johnston, 2002; Takei & Nishida, 2010). Response times, however, might reflect speedy decisions that can occur prior to perception or rely on different neural circuits than perception (Neumann & Niepel, 2004).

*ΔL* is often estimated from other behavioural tasks that are considered to rely more upon perception such as temporal order judgments (TOJ) (Adams & Mamassian, 2004; Arnold, Johnston, & Nishida, 2005; Aschersleben & Musseler, 1999; Barnett-Cowan & Harris, 2009; Bedell, Chung, Ogmen, & Patel, 2003; Di Luca et al., 2009; Johnston, Arnold, & Nishida, 2006; Kanai, Carlson, Verstraten, & Walsh, 2009; Klemm, 1925; Kopinska & Harris, 2004; Lewald & Guski, 2004; Linares & López-Moliner, 2006; Neumann & Niepel, 2004; Sugita & Suzuki, 2003), simultaneity judgments (SJ) (Barnett-Cowan & Harris, 2009; Kanai et al., 2009; Stone et al., 2001) and duration judgments (Kanai & Watanabe, 2006; Mayer, Di Luca & Ernst, 2014). Here, we studied TOJ and SJ because they appear to be the most commonly used for estimating *ΔL*. We also used duration judgments because, although previously it has rarely been used to estimate *ΔL*, the large temporal interval that can be used between stimulus *A* and *B* should minimize or eliminate the sensory interaction of *A* and *B* that may be a problem for the other tasks (see Discussion).

In TOJ (Hirsh & Sherrick, 1961), *A* and *B* are presented with different relative timings and observers report which stimulus occurred first. *ΔL* is estimated as the relative timing for which it is equally likely to report *A* is first as that *B* is first.

In SJ (Allan, 1975), *A* and *B* are presented with different relative timings and observers report whether they occurred at the same time or not. *ΔL* is estimated as the relative timing that is most likely to cause an observer to report “simultaneous.”

In duration judgments (Grondin & Rousseau, 1991; Kanai & Watanabe, 2006; Mayer, Di Luca & Ernst, 2013), observers compare the duration of an interval delimited by *A* followed by *B* (*AB*) with the duration of an interval delimited by *B* followed by *A* (*BA*). If the perceptual latency of *A* (*L*_*A*_) is shorter than the perceptual latency of *B* (*L*_*B*_), then the interval *AB* should be perceived as lasting longer than *BA* (Figure 3A; Kanai & Watanabe, 2006; Mayer, Di Luca & Ernst, 2013). More specifically, the perceived duration of an interval *AB* (*D*_*AB*_) of physical duration *d* might be estimated as

(1) $$D_{\mathrm{AB}}=d+\Delta L_{\mathrm{BA}},$$

where *ΔL*_*BA*_ = *L*_*B*_ − *L*_*A*_ (Kanai & Watanabe, 2006; Mayer, Di Luca & Ernst, 2013).

Similarly, the perceived duration of a *BA* interval (*D*_*BA*_) of the same duration might be estimated as

(2) $$D_{\mathrm{BA}}=d-\Delta L_{\mathrm{BA}}.$$

Hence, from Equations (1) and (2) it is possible to estimate *ΔL*_*BA*_ using *D*_*AB*_ and *D*_*BA*_

(3) $$\Delta L_{\mathrm{BA}}=1/2(D_{\mathrm{AB}}-D_{\mathrm{BA}}).$$

A major problem for the estimation of *ΔL* is that the estimates provided by different tasks often do not coincide. For TOJ and SJ, previous studies have shown that *ΔL* are inconsistent in that they do not correlate significantly across observers (Love, Petrini, Cheng, & Pollick, 2013; van Eijk, Kohlrausch, Juola, & Van de Par, 2008; Vatakis, Navarra, Soto-Faraco, & Spence, 2008; but see Sanders, Chang, Hiss, Uchanski, & Hullar, 2011). This inconsistency might be related to the different biases that afflict TOJ and SJ (see Discussion).

No previous studies have tested whether the *ΔL* estimated from SJ or TOJ is consistent with that estimated from duration judgments. Consistency of duration judgments and TOJ might suggest that these methods should be preferred for estimating *ΔL* (Neumann & Niepel, 2004). Another possibility is that SJ and duration judgments yield consistent estimates. We found, however, that *ΔL* estimated from duration judgments is not consistent with *ΔL* estimated from TOJ nor SJ, which suggests that each method is subject to different processes that hinder the estimation of *ΔL*.

## 2 Method

Seven people participated. The authors (DL and AH) and GS knew the experimental hypotheses. The tasks of duration judgment, SJ, and TOJ were tested in different sessions. Observers conducted first the duration judgments, then the SJ and finally the TOJ. The sole exception was AH, who conducted some more sessions of duration judgments after the TOJ. For the duration judgments, two standard durations (see below) were tested in different sessions whose order was pseudo-randomized across observers. For each standard, observers completed between 280 and 1,120 trials. For SJ and TOJ, each observer completed 220 trials. The data and code (in R, R Core Team, 2014) to do the statistical analysis and create the figures are available at http://www.dlinares.org.

The visual and auditory stimuli were generated using PsychoPy (Peirce, 2007). Visual stimuli were displayed on a CRT at 100 Hz and auditory stimuli were presented with headphones. Observers fixated a black circle (all guns set to zero) in a grey background (54 cd/m^2^). The visual event, *V*, was a colour change of the fixation point to white (108 cd/m^2^) for 10 ms. The auditory event, *A*, was a 10 ms white noise burst (70 dB SPL).

For SJ and TOJ, *A* occurred at a random time between 0.8 and 1.2 s after the onset of the trial and the timing of *V* relative to *A* varied according to the method of constant stimuli (ranging from −0.25 to 0.25 s in steps of 0.05 s). SJ and TOJ were run in separate blocks of trials.

For the duration judgments, two intervals were presented on each trial and the observers judged which was longer (see Figure 3a). The first interval (the “standard”) was 0.6 s or 1.2 s in different blocks of trials. The second (the “variable”) was chosen from a range of durations centred on the standard interval's duration (method of constant stimuli; variable intervals for the 0.6 s standard: 0.3, 0.4, 0.5, 0.6, 0.8 s; variable intervals for the 1.2 s standard: 1.0, 1.2, 1.6, 2.0, 2.4 s). The irrelevant “spacing interval” between the judged intervals had a random duration between 0.4 and 0.6 s for the 0.6 s standard, and between 0.8 and 1.2 s for the 1.2 s standard. The time preceding the stimuli on each trial was a random value between 0.4 and 0.6 s for the 0.6 s standard and a random value between 0.8 and 1.2 s for the 1.2 s standard.

## 3 Results

### 3.1 Temporal order judgments (TOJ)

**Figure 1.**
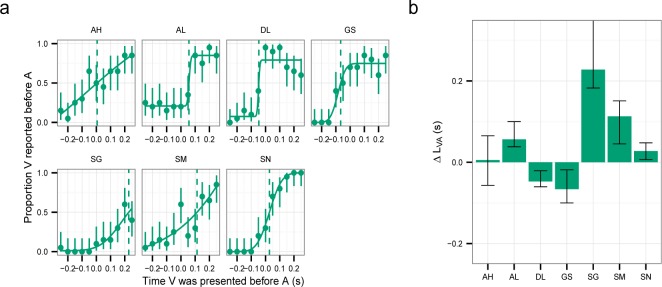
Temporal order judgments (TOJ). (a) Proportion of *V* reported before *A* as a function of the relative timing between *A* and *V*. The error bars are the 95% confidence intervals calculated using the Clopper–Pearson method. The four-parameter (mean, standard deviation, lower asymptote and upper asymptote) cumulative normal distributions were fitted using maximum likelihood estimation (Knoblauch & Maloney, 2012). (b) *ΔL*_*VA*_ estimated as the timing for which half of the trials the observer reported *V* before *A* in (a). Positive values indicates longer latency for *V*. The error bars are the 95% parametric bootstrap confidence intervals (Kingdom & Prins, 2010; we use 1,000 samples for all the bootstrap calculations in the paper).

Figure 1a shows the proportion of trials in which *V* (the visual stimulus) was reported to occur before *A* (the auditory stimulus) as a function of the relative timing between *A* and *V*. Many observers informally reported that this task was more difficult than the SJ task and the duration task. The participants in Love et al. (2013) similarly reported that TOJ was more difficult. We found that some observers like GS and DL perform far from perfectly (less than 80% correct) even for very long relative timings, so it appears that the greater difficulty of TOJ is not restricted to when the stimuli occur close in time. To account for this, we fitted cumulative normal curves with an independent lapse rate for each side (see caption of Figure 1).

To estimate *ΔL*_*VA*_ from the fitted curves, we extracted the timing for which in half of the trials the observer reported *V* before *A*. This *ΔL*_*VA*_ is positive for some observers and negative for others, which is statistically reliable as for most observers the confidence intervals do not overlap zero (Figure 1b). This is consistent with previous findings (Boenke, Deliano, & Ohm, 2009; Love et al., 2013; van Eijk et al., 2008). Across observers, the average *ΔL*_*VA*_ was 45 ms (not significantly different from 0, one sample *t*-test: *t*(6) = 1.89, *p* = .28).

### 3.2 Simultaneity judgments (SJ)

**Figure 2.**
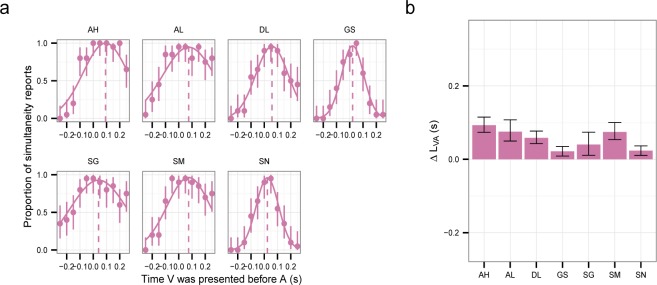
Simultaneity judgments (SJ). (a) Proportion of simultaneity reports. The error bars are the 95% confidence intervals calculated using the Clopper–Pearson method. The three-parameter (amplitude, mean and standard deviation) normal distributions were fitted using maximum likelihood estimation. (b) ΔLVA estimated as the mean of the fitted distribution in (a). Positive values indicates longer latency for *V*. The error bars are the 95% bootstrap parametric confidence intervals.

Figure 2a shows the proportion of simultaneous reports as a function of the relative timing between *A* and *V*. We fitted a normal curve to these proportions and took its center as *ΔL*_*VA*_ (see caption of Figure 2). *ΔL*_*VA*_ was positive for all observers (their confidence intervals do not include zero, Figure 1b). This suggests faster perception of *A* relative to *V* and is consistent with the findings of previous studies that most observers' *ΔL*_*VA*_ is positive (Arrighi, Alais, & Burr, 2006; Love et al., 2013; Stone et al., 2001; van Eijk et al., 2008; Zampini, Guest, Shore, & Spence, 2005;). Across observers, the average *ΔL*_*VA*_ was 55 ms (significantly different from 0, one sample *t*-test: *t*(6) = 5.31, *p* = .002).

### 3.3 Duration judgments

**Figure 3.**
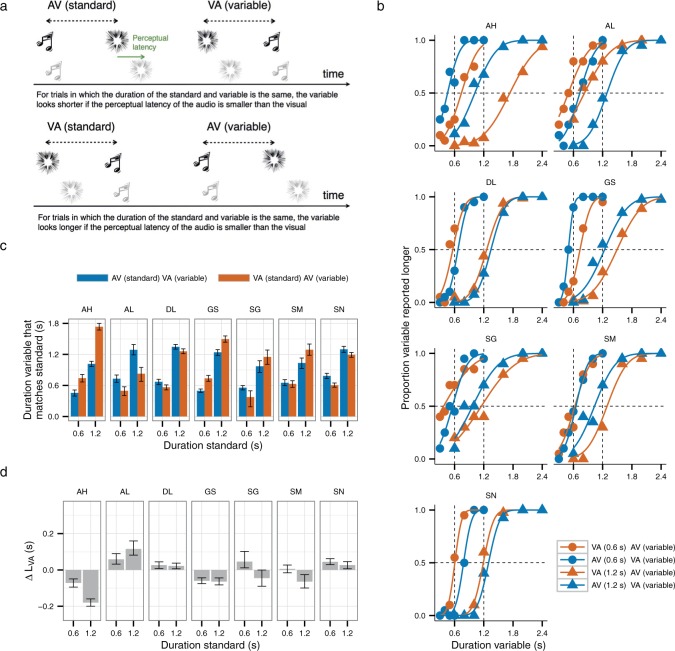
Duration judgments. (a) Illustration of the two type of trials used. (b) Proportion of trials in which the variable was perceived as lasting longer than the standard for the two types of trials and the two standards. The cumulative normal distributions were fitted using maximum likelihood estimation. (c) Duration variable that matches the standard from (a), PSE. The error bars are the 95% confidence intervals calculated by parametric bootstrap (d) Differences in perceptual latency for auditory and visual stimulation, *ΔL*_*VA*_, calculated as the difference in the PSEs for the two types of trials in (c) divided by 4 (Equation (4)). Positive values indicates longer latency for *V*. The error bars are the 2.5 and 97.5 percentiles of the upper and lower limits of the distribution of differences of the bootstrapped PSEs in (c) for the two type of trials divided by 4.

In each trial, the observers decided which of two intervals bounded by *A* and *V* seemed longer (Figure 3a). The order of presentation of *A* and *V* was different in two distinct trial types. In the *AVVA* type of trial, the first interval was a 0.6 s “standard”: *A* was always followed by *V* (standard *AV*) after 0.6 s. The second interval was *V* followed by *A* after a variable amount of time (variable *VA*). In the *VAAV* type of trial, the first interval was a standard of 0.6 s in which *V* was followed by *A* (standard *VA*) and the second interval was defined by *A* followed by *V* after a variable amount of time (variable *AV*). Thus, the standard was always presented before the variable. The *AVVA* and *VAAV* trial types were presented in random order within each session.

Comparison of the PSEs for the two trial types allows estimation of *ΔL*_*VA*_, as explained in the Introduction. The difference in perceptual latency between visual and auditory stimulus, *ΔL*_*VA*_, should not depend on the duration of the interval demarcated by the two stimuli, *d* (Mayer, Di Luca & Ernst, 2013), which here is formalized in Equation (3). To test this, in different blocks we used two different durations of the standard, 0.6 s and 1.2 s.

Figure 3b shows, for each observer and standard, the proportion of trials in which the variable interval was perceived to last longer than the standard for *AVVA* and *VAAV* trials. Figure 3c shows for *AVVA* and *VAAV* trials the durations of the variable intervals that match the standards. These durations are the points of subjective equality or PSEs, estimated by fitting cumulative normal curves to the data in Figure 3b and taking from these fits the duration of the variable intervals that were reported longer than the standards in half of the trials.

Figure 3d shows the estimated *ΔL*_*VA*_ values. We did not estimate *ΔL*_*VA*_ by applying Equation (3) directly to the PSEs in Figure 3c because the equations in the Introduction assume that perceived duration is measured using a “neutral” variable interval (Mayer, Di Luca & Ernst, 2013). Instead of using neutral variable intervals, we used variable intervals in which the order of *A* and *V* was reversed relative to the standards because this yields twice the expected effect, providing more power to reveal differences in the perceived duration between *AV* and *VA* intervals. Taking into account the use of reversed variable intervals when considering Equations (1), (2) and (3) yields the following equations:

(4) $$D_{\mathrm{AV}}=d+2\Delta L_{\mathrm{VA}},$$

(5) $$D_{\mathrm{VA}}=d-2\Delta L_{\mathrm{VA}},$$

(6) $$\Delta L_{\mathrm{VA}}=1/4(D_{\mathrm{AV}}-D_{\mathrm{VA}}).$$

Hence, we estimated *ΔL*_*VA*_ by subtracting the PSEs shown in Figure 3c and dividing them by 4.

Before discussing the *ΔL*_*VA*_ values, we should point out that, according to Equations (4) and (5), the deviation of the PSE for *AVVA* relative to the duration of the standard (*D*_AV_ − *d*) and the deviation of the duration of the standard relative to the PSE for *VAAV* (*d* − *D*_*VA*_) should be equal. These quantities, however, were not equal for 8 of the 14 samples (7 observers × 2 standards), according to a statistical test. The statistical test was calculation of the 95% confidence intervals of (*D*_AV_ − *d*) − (*d* − *D*_*VA*_) for each bootstrapped within-subject PSE sample and checking whether it contained zero. Between-subjects statistics support the same conclusion for the population generally—that the deviation is asymmetric. This was a one-sample *t*-test using the absolute values of (*D*_AV_ − *d*) − (*d* − *D*_*VA*_) calculated for each observer. This difference in shift of the perceived duration relative to *d* was statistically significant for the 1.2 s standard (*t*(6) = 5.50, *p* = .002) indicating asymmetry, and marginally so for the 0.6 s standard (*t*(6) = 2.40, *p* = .05).

The asymmetric shift could be caused by a bias to perceive the first interval as shorter or as longer than the second interval (time order error, Hellström, 1985). To take into account this effect, one can consider *d* in Equations (4) and (5) not as the physical interval but the interval plus a constant term corresponding to shortening or lengthening. Fortunately, because Equation (6) is obtained by subtracting Equations (4) and (5), thus removing *d*, the shortening or lengthening of the first interval should not affect the calculation of *ΔL*.

Figure 3d shows the estimated *ΔL*_*VA*_ for the 0.6 and 1.2 s standards. For each observer, the sign of *ΔL*_*VA*_ was consistent across standards, that is, for any observer *ΔL*_*VA*_ was never significantly positive for one of the standards and significantly negative for the other (confidence intervals in Figure 3d). Furthermore, the estimated *ΔL*_*VA*_ for the two standards correlated across observers: Pearson's correlation, *r*(5) = .82, *p* = .02, 95% bootstrap CI = (.43, .98). Across observers, *ΔL*_*VA*_ also was not significantly different for the 0.6 s and 1.2 s standards (paired *t*-test, *t*(6) = 1.55, *p* = 0.17). These results seem consistent with Equation (3). They are also consistent with a recent study finding no effect of the standard's duration, although that study did not examine the issue as closely because the data were pooled across observers before conducting the statistical analysis (Mayer, Di Luca & Ernst, 2013).

At the individual observer level, however, the data do not support the consistency across standards. For four of the seven observers, the estimated *ΔL*_*VA*_ for the 0.6 s and the 1.2 s standards were different on the conservative test (*p* < .006) of non-overlap of the confidence intervals (Knol, Pestman, & Grobbee, 2011), which suggests that *ΔL*_*VA*_ estimates are inconsistent, even here where the task (duration judgments) was the same for the two conditions, and only the magnitude of the interval differed.

### 3.4 Comparison across tasks

**Figure 4.**
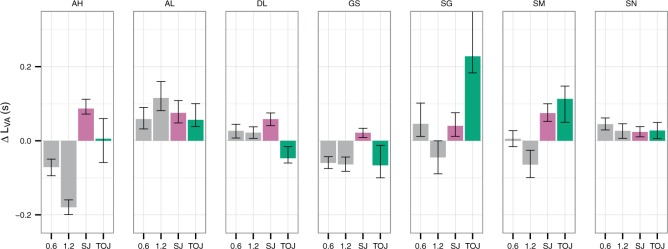
Replot of the estimated *ΔL*_*VA*_ from Figures 1d, 2b and 3b.

Figure 4 compares the *ΔL*_*VA*_ estimated by the different methods for each observer. Just as previous studies have found (Love et al., 2013; van Eijk et al., 2008; Vatakis et al., 2008; but see Sanders et al., 2011), *ΔL*_*VA*_ estimated from TOJ and SJ did not correlate across observers: *r*(5) = .078, *p* = .87, 95% CI = (−.81,.87). At the individual level, for four of the seven observers the estimated *ΔL*_*VA*_ from TOJ and SJ were different as indicated by the non-overlap of the confidence intervals.

For duration judgments, the *ΔL*_*VA*_ correlated neither with the *ΔL*_*VA*_ from TOJ nor with that of SJ: duration for 0.6 s standard vs TOJ, *r*(5) = .49, *p* = .26, CI = (−.09,.89); duration for 0.6 s standard vs SJ, *r*(5) = −.16, p = .73, CI = (−.93,.82); duration for 1.2 s standard vs TOJ, *r* = .0086, *p* = .99, CI = (−.80,.68); duration for 1.2 s standard vs SJ, *r*(5) = −.24, *p* = .61, 95% CI = (−.92, .87). At the individual level—judging by the non-overlap of the confidence intervals—for four of the seven observers *ΔL*_*VA*_ from the 0.6 s standard and TOJ were different; for four of the seven observers *ΔL*_*VA*_ from the 0.6 s standard and SJ were different; for four of the seven observers *ΔL*_*VA*_ from the 1.2 s standard and TOJ were different; for five of the seven observers *ΔL*_*VA*_ from the 1.2 s standard and SJ were different.

Although *ΔL*_*VA*_ for the two standards correlated across observers, the lack of correlation for the other comparison might not be very informative because our study did not include many participants. We collected data for a small sample because we were interested in the consistency of *ΔL*_*VA*_ at individual level. Our results reveal strong inconsistencies in more than half of the observers.

Future studies using larger samples of participants might establish which tasks produce estimates of *ΔL*_*VA*_ that are less inconsistent. Larger samples could also reveal which tasks produce estimates of *ΔL*_*VA*_ that are less variable across participants.

## 4 Discussion

Using an auditory and a visual stimulus, we showed that *ΔL* estimated from SJ and TOJ are inconsistent, which accords with previous studies (Love et al., 2013; van Eijk et al., 2008; Vatakis et al., 2008; but see Sanders et al., 2011) and found that *ΔL* estimated from duration judgments is consistent with neither TOJ nor SJ. Given these inconsistencies, it is difficult to know which task is best for estimating *ΔL*. In Table 1, we offer a tentative list of the possible problems associated with using each task to estimate *ΔL*. We discuss these problems below.

**Table 1.** Problems estimating *ΔL*. “Yes” indicates that the task is likely affected by the problem.

Problem	TOJ	SJ	Duration
Sensory interactions	Yes	Yes	No
Method of single stimuli	Yes	No	No
Temporal order difficulty	Yes	No	No
Asymmetric criterion	No	Yes	No
Duration order	No	No	Yes

### 4.1 Problems estimating *ΔL* from TOJ

Estimating *ΔL* from TOJ may be affected by three problems. First, sensory interactions: in TOJ, the stimuli are presented close in time and that can affect the response of sensory neurons to each (e.g. temporal principle in multisensory processes, Meredith, Nemitz, & Stein, 1987), altering their neural latencies (e.g. Rowland, Quessy, Stanford, & Stein, 2007). Thus, the perceptual latency of *A* presented alone and the perceptual latency of *A* when presented near *V* could be different. For example, when *A* and *V* are presented close in time, attention might be allocated differently to *A* and *V*. Given that attending to a stimulus can reduce its neural latency by a few milliseconds (Galashan, Saßen, Kreiter, & Wegener, 2013; Sundberg, Mitchell, Gawne, & Reynolds, 2012), the different allocation of attention might introduce differences in perceptual latency—an effect called prior entry (Spence & Parise, 2010).

The second problem is a bias associated generally with the method of single stimulus or comparative judgments—a popular method for measuring appearance (Anton-Erxleben, Abrams, & Carrasco, 2010; García-Pérez & Alcalá-Quintana, 2013). TOJ is an instance of the method of single stimulus and as such its estimates may be contaminated by a response bias (Anton-Erxleben et al., 2010; Arnold et al., 2005; García-Pérez & Alcalá-Quintana, 2012; García-Pérez & Alcalá-Quintana, 2013; Jazayeri & Movshon, 2007; Morgan, Dillenburger, Raphael, & Solomon, 2012; Nicholls, Lew, Loetscher, & Yates, 2011; Schneider & Bavelier, 2003; Shore, Spence, & Klein, 2001; Yarrow, Jahn, Durant, & Arnold, 2011). This bias is particularly an issue for temporal asynchronies near the PSE, where the observer might frequently feel that she does not have an answer, but because she needs to respond, she ends up favouring one of the responses or response buttons.

Favouring one response button means that under uncertainty the participant might have a preference, for example, for the button that she presses with the index finger or with the dominant hand instead of choosing between the buttons completely at random.

Favouring one response might mean, for example, choosing rightward direction in a leftward/rightward motion discrimination task (Morgan et al., 2012), or the auditory stimulus in a TOJ task. The reasons for choosing one response over the other might not be obvious and different observers might have different preferences. In other cases, the bias might be fairly consistent across observers. For example, in a TOJ task, observers might report that attended stimuli come earlier when they are uncertain about the response. Indeed, it is possible that this bias is the major contribution to the prior entry effect (Matthews, Welch, Festa, & Clement, 2013; Reeves & Sperling 1986; Shore et al., 2001; Schneider & Bavelier, 2003; Spence & Parise, 2010). A response could also be favoured depending on how the stimuli group responds. When three stimuli are presented during a brief interval, for example, the reported order of the last two might depend on the similarity with the first one (Albertazzi, 1999; Holcombe, in press; Koenderink, Richards, & Doorn, 2012; Sinico, 1999).

The third problem is a bias associated with a difficulty ordering events in time. In the method of single stimuli for spatial tasks, such as motion discrimination, most observers give the correct response for stimuli relatively far from the PSE, e.g., they respond “rightward” close to 100% of trials if the motion signal is strongly to the right (e.g. Selen, Shadlen, & Wolpert, 2012). For TOJ however, our observers, like observers in previous studies (Love et al., 2013; Petrini, Holt, & Pollick, 2010; Zampini, Shore, & Spence, 2003), have numerous “lapses” even for relatively large temporal separation of the two stimuli. This poor performance is consistent with the data of our observers and observers in previous studies (e.g. Love et al, 2013) reporting that TOJ was a difficult task, more so than SJ. The difficulty might be related to a cognitive temporal bottleneck that limits mapping the two stimuli to their identity (Yamamoto & Kitazawa, 2001). That is, not having enough time to name each stimulus before the next one is perceived can leave one unable to do the task. Depending on the curve used to fit the data (García-Pérez & Alcalá-Quintana, 2012), the numerous lapses far from the PSE may contaminate the estimation of the PSE (García-Pérez & Alcalá-Quintana, 2012), sometimes even precluding its estimation (Love et al., 2013; Petrini et al., 2010; Zampini et al., 2003).

### 4.2 Problems estimating *ΔL* from SJ

Given that in SJ, like in TOJ, stimuli are presented close in time, sensory interactions might also afflict SJ. SJ is an instance of the method of equality judgments that measure appearance (Anton-Erxleben et al., 2010; García-Pérez & Alcalá-Quintana, 2012). In equality judgments, the bias associated with the method of single stimuli should not occur because favouring one of the responses or response-buttons should change the height of the fitted curve but should not shift its central tendency, the PSE (Anton-Erxleben et al., 2010; García-Pérez & Alcalá-Quintana, 2012; Schneider & Bavelier, 2003).

Equality judgments, however, might be affected by an asymmetrical criterion bias by which the probability to report “equal” or “simultaneous” is different for stimuli smaller and larger than the PSE (Anton-Erxleben et al., 2010; Yarrow et al., 2011). For SJ, this bias might reflect an asymmetrical criterion to perceive two stimuli as related. It has been suggested that for auditory and visual stimuli, observers perceive auditory following visual to be more likely to be related than the reverse order, possibly because the former is more common in the environment (Dixon & Spitz, 1980).

Unfortunately, observers sometimes report not having a clear sense of whether two stimuli were simultaneous (Hirsh & Fraisse, 1964; Klemm, 1925). Especially in such conditions of uncertainty, judgments may instead be based on perceived relatedness, which can have an asymmetrical criterion (Welch & Warren, 1980). Indeed, SJ is often considered a measure of perceptual integration that depends on other things as well as *ΔL* (Arrighi, Alais, & Burr, 2006; Love et al., 2013; Powers, Hillock, & Wallace, 2009; Sanders et al., 2011; van Eijk et al., 2008; Zampini et al., 2005;).

Consistent with the hypothesis of an asymmetrical criterion that favours judging simultaneity for auditory stimuli occurring after visual stimuli, three recent studies suggest that perceived simultaneity is easily malleable for the situation in which *A* follows *V* but not for the situation in which *V* follows *A*. The first study found that short training on SJ for *A* and *V* with feedback reduces “simultaneity” reports for *A* following *V*, but not for *V* following *A* (Powers et al., 2009). The second study reported that video game players report fewer “simultaneous” responses when *A* follows *V* than non-video game players, but video game and non-video game players report “simultaneous” equally when *V* follows *A* (Donohue, Woldorff, & Mitroff, 2010). The third found that fast adaptation to temporal relationships occurs when *A* follows *V*, but not when *V* follows *A* (van der Burg, Cass, & Alais, 2013). It is possible, however, that these changes are not just decisional but reflect actual changes in perceptual latency.

To try to reconcile the differing estimates of *ΔL* from TOJ and SJ, the bias associated with the method of single stimuli has been modeled recently by García-Pérez and Alcalá-Quintana (Alcalá-Quintana & García-Pérez, 2013; García-Pérez & Alcalá-Quintana, 2012), but when we did a simple Pearson-product correlation of their estimates of *ΔL* for TOJ and SJ, we found that *ΔL* did not correlate across observers (*r*(9) = .23, p = .50; table 1 of the Appendix 2 in García-Pérez & Alcalá-Quintana, 2012). The problem might be that their model does not incorporate any bias associated with the difficulty to order events for TOJ nor the asymmetrical criterion bias for SJ. The former might be easy to incorporate in the form of lapses (García-Pérez & Alcalá-Quintana, 2012), but not the latter given that it requires knowledge about how observers associate relatedness to events.

### 4.3 Problems estimating *ΔL* from duration judgments

Because the stimuli in the duration task can be presented far apart in time (as they were here), the duration task may avoid the problem of sensory interactions and also the bias associated with temporal order difficulty.

We measured duration judgments using a two-interval forced choice method. In principle, this method should not be affected by the bias associated with the method of single stimuli. Unfortunately, however, we presented the standard interval always first, and hence, when observers reported shorter or longer for the second interval, they were effectively prone to the same bias as that with the method of single stimuli. The bias could be avoided, if the order of presentation of the standard is randomized.

Another bias that may apply only to the duration task is something we refer to as the duration order bias (Kanai & Watanabe, 2006; Mayer, Di Luca & Ernst, 2013). The derivation of Equation 3 assumes than the order of presentation of the stimuli that delimit the time interval only affects the perceptual latency of the onset and offset of the interval and not the encoding of duration per se (Ivry & Schlerf, 2008; Kanai & Watanabe, 2006; Mayer, Di Luca & Ernst, 2013). Under this assumption, *ΔL* should not depend on the duration of the interval, but instead we found that it does, which suggests that the order of presentation of the stimuli affects the encoding of duration per se and that duration judgments are problematic to estimate *ΔL*. Such a duration order bias might occur if, for example, attention is prompted differently at the interval onset by *A* and *V* (see Discussion in Kanai & Watanabe, 2006) given that attention has been shown to influence perceived duration (Tse, Intriligator, Rivest, & Cavanagh, 2004).

## 5 Conclusions

The inconsistent estimates of *ΔL* for TOJ, SJ and duration judgments suggest that these tasks are affected by partially distinct sets of biases. Hence, when researchers use only one of these tasks to assess the effect on *ΔL* of some manipulation—such as attention (Spence & Parise, 2010), temporal context (Chen & Vroomen, 2013; Koenderink et al., 2012) or adaptation (Fujisaki, Shimojo, Kashino, & Nishida, 2004)—any significant effect should be attributed to changes in *ΔL* only if one is confident that the biases did not change.

To evaluate the merits of different tasks in the estimation of *ΔL*, one might not only consider the consistencies of *ΔL* across tasks, but also the consistency of *ΔL* for an individual across different moments in time. This has not been done extensively, but for SJ, previous results suggest that the estimated *ΔL* is stable (Stone et al., 2001).

Apart from TOJ, SJ and duration judgments, other tasks can also be used to estimate perceptual latencies. Observers might be asked to report the location of a moving object at the time of an event (Haggard, Clark, & Kalogeras, 2002; Kanai, Sheth, Verstraten, & Shimojo, 2007; Libet, Gleason, Wright, & Pearl, 1983; Linares, Holcombe, & White, 2009; Wundt, 1883) or synchronize a motor action with a sensory event (Aschersleben & Musseler, 1999; Linares et al., 2009; Nishida & Johnston, 2002; Repp & Su, 2013; White, Linares, & Holcombe, 2008). There are also variations of the duration task in which participants attempt to press a button for the same duration as a previous sensory event (Jazayeri & Shadlen, 2010), or judge whether an event occurred exactly at the midpoint of an interval (Burr, Banks, & Morrone, 2009). Differences in perceptual latencies have also been estimated by asking participants to determine the pairing of two alternating features (Clifford, Arnold, & Pearson, 2003; Moutoussis & Zeki, 1997), although the notion that the best feature timing reflects the difference in perceptual latency has been questioned (Holcombe & Cavanagh, 2008; Holcombe, 2009; but see Moutoussis, 2012; Nishida & Johnston, 2002). The consistency of latencies inferred from these tasks with those from others is under-studied.

To provide stronger support for a conclusion that a manipulation affected *ΔL* rather than just the biases, researchers should consider using multiple tasks. If the perceptual latency estimates from all tasks support the same conclusion regarding a change in *ΔL*, the conclusion is more secure. After at least some manipulations, however, the changes in latencies inferred from different tasks disagree (e.g. Schneider & Bavelier, 2003; Vatakis et al., 2008), suggesting that change in biases are sometimes the cause of apparent *ΔL* shifts. More work should be done to elucidate the nature and malleability of these biases.
